# Levels of Structural Integration Mediate the Impact of Metacognition on Functioning in Non-affective Psychosis: Adding a Psychodynamic Perspective to the Metacognitive Approach

**DOI:** 10.3389/fpsyg.2020.00269

**Published:** 2020-02-21

**Authors:** Anna-Lena Bröcker, Samuel Bayer, Frauke Stuke, Sandra Just, Gianna Bertram, Jakob Funcke, Imke Grimm, Günter Lempa, Dorothea von Haebler, Christiane Montag

**Affiliations:** ^1^Department of Psychiatry and Psychotherapy, Charité Universitätsmedizin Berlin, Berlin, Germany; ^2^International Psychoanalytic University Berlin, Berlin, Germany; ^3^Psychotherapy Practice, Munich, Germany

**Keywords:** psychic structure, internalized object representations, Operationalized Psychodynamic Diagnosis, synthetic metacognition, MAS-A, mentalizing abilities, psychotherapy of psychosis, psychosocial functioning

## Abstract

Synthetic metacognition is defined by integrative and contextualizing processes of discrete reflexive moments. These processes are supposed to be needed to meet intrapsychic as well as interpersonal challenges and to meaningfully include psychotic experience in a personal life narrative. A substantial body of evidence has linked this phenomenon to psychosocial functioning and treatment options were developed. The concept of synthetic metacognition, measured with the Metacognition Assessment Scale-Abbreviated (MAS-A), rises hope to bridge gaps between therapeutic orientations and shares valuable parallels to modern psychodynamic constructs, especially the ‘levels of structural integration’ of the Operationalized Psychodynamic Diagnosis (OPD-2). As theoretical distinctions remain, aim of this study was to compare the predictive value of both constructs with regard to psychosocial functioning of patients with non-affective psychoses, measured with the International Classification of Functioning, Disability and Health (MINI-ICF-APP). It was further explored if levels of structural integration (OPD-LSIA) would mediate the impact of metacognition (MAS-A) on function (MINI-ICF-APP). Expert ratings of synthetic metacognition (MAS-A), the OPD-2 ‘levels of structural integration’ axis (OPD-LSIA), psychosocial functioning (MINI-ICF-APP) and assessments of general cognition and symptoms were applied to 100 individuals with non-affective psychoses. Whereas both, MAS-A and OPD-LSIA, significantly predicted MINI-ICF-APP beyond cognition and symptoms, OPD-LSIA explained a higher share of variance and mediated the impact of MAS-A on MINI-ICF-APP. Levels of structural integration, including the quality of internalized object representations and unconscious interpersonal schemas, might therefore be considered as valuable predictors of social functioning and as one therapeutic focus in patients with non-affective psychoses. Structural integration might go beyond and form the base of a person’s actual reflexive and metacognitive capabilities. Psychotherapeutic procedures specific for psychoses may promote and challenge a patient’s metacognitive capacities, but should equally take the need for maturing structural skills into account. Modern psychodynamic approaches to psychosis are shortly presented, providing concepts and techniques for the implicit regulation of interpersonal experience and aiming at structural integration in this patient group.

## Introduction

As psychotherapeutic treatment options for individuals with psychotic disorders are growing, many of them focus on metacognition as one potential target of intervention. Interventions differ as they start from a varying understanding of the term “metacognition” (dating back to Flavell, 1979) that is rather broad and needs clarification (Brüne, 2014; Moritz and Lysaker, 2018). It can be differentiated between *discrete* metacognitive acts, thus general knowledge and awareness of cognitions that enable an individual to control, monitor and reflect upon habitual cognitive processes, and more *synthetic* metacognitive acts, in which discrete knowledge about self and others in terms of mental states (cognition, emotions, intentions) is continuously integrated into larger representations (Lysaker et al., 2013b). On the continuum from a discrete to a synthetic understanding of the term, discrete interventions that focus on fostering an active control of general dysfunctional metacognitive strategies such as rumination (Morrison et al., 2014, 2015) exist along with manualized group (Moritz and Woodward, 2007) and individual trainings (Moritz et al., 2011) that aim at creating an awareness of specific cognitive biases that were identified as characteristic for productive psychotic symptoms (Garety and Freeman, 2013). Synthetic metacognition on the other hand ranges from the identification of discrete emotional or cognitive mental states to higher-order reflexive and integrative processes (Lysaker and Klion, 2017; Lysaker et al., 2018b). Therapeutic approaches that step in here (Rosenbaum et al., 2012; Salvatore et al., 2012; Lempa et al., 2016; Lysaker and Klion, 2017; Schweitzer et al., 2017) do not necessarily address specific symptom dimensions (Lysaker and Dimaggio, 2014), but foster the ability to generate coherent narratives as well as to contextualize experiences with regard to a subject-oriented recovery process (Kukla et al., 2013; Klapheck et al., 2014).

Synthetic metacognition implies semi-independent sub-functions (Semerari et al., 2003; Dimaggio et al., 2008), like self and other reflection, decentration or the capacity to change perspective, and mastery as the ability to use this knowledge (in terms of cognitive acts, emotions as well as their intercorrelations) in order to meet intrapsychic as well as interpersonal challenges. These sub-functions were initially integrated in the Metacognition Assessment Scale (MAS; Semerari et al., 2003, 2007), a tool to assess frequency of metacognitive acts within psychotherapy transcripts. Its adaption, the Metacognition Assessment Scale-Abbreviated (MAS-A; Lysaker et al., 2005, 2010a), was then applied to interview situations with individuals diagnosed with psychosis. Though metacognition gained more attention in the field of cognitive behavioral therapy, it is – in its synthetic understanding – close to popular psychodynamic constructs, especially mentalizing (Fonagy et al., 2002; Brent, 2009; Dimaggio and Lysaker, 2015) and might thus provide a common language and help to bridge the gap between different therapeutic orientations.

Possible origins of reduced metacognitive abilities were investigated, including reports of early experiences, specifically childhood trauma (Aydin et al., 2016; Lysaker and Klion, 2017; Weijers et al., 2018). However, healthy as well as pathological early shaped interpersonal schemas can – from a psychoanalytic viewpoint – be implicit and unconscious. They might strongly influence actual perceptions of self, others and mastery of the social world’s challenges. However, conceptualizations of synthetic metacognition do so far not encompass the notion of these internalized interaction experiences and implicitly working inner object relations and may therefore be enriched by the psychoanalytic perspective (Lysaker et al., 2010a; Hamm and Lysaker, 2016).

“Psychic structure” as a psychodynamic key concept has a long tradition (e.g., Abraham, 1925; Bellak and Hurvich, 1969; Kohut, 1971; Kernberg, 1975, 1984; Rudolf, 1977; Shapiro, 1991) and describes a broader array of inner-psychic core competencies or “ego-functions” that are unconsciously activated in everyday social interactions and in light of critical biographic incidents in order to sustain intrapsychic balance and interpersonal function. With the aim to integrate different theoretical perspectives (Rudolf et al., 1995; Cierpka et al., 2007), important dimensions of psychic structure were summarized in the Operationalized Psychodynamic Diagnosis system (OPD-2; OPD-2 Task Force, 2014), precisely in one out of five axes called “level of structural integration” (OPD-LSIA). It comprises abilities of the reflexive perception concerning the self and others, regulation of the self and object-relationships, internal communication vs. communication with the external word and attachment capacities to internal and external objects. While the concept of synthetic metacognition focuses on an increasingly complex knowledge of self and others that helps to master and learn from psychological challenges, OPD-LSIA evaluates the underlying experiences of early attachment, the development of inner object relations and the unconscious influence of internalized interpersonal interactions as important predictors of self-regulation and social functioning.

However, psychic structure has not gained much attention in systematic research of non-affective psychoses (Uzdawinis et al., 2010) and a potential association with psychosocial functioning has, best to our knowledge, never been investigated in this patient group. Improved synthetic metacognitive abilities, on the other hand, have already been associated with increased functioning across different studies including first and multiple psychotic episodes (meta-analysis by Arnon-Ribenfeld et al., 2017; Gagen et al., 2019). Lysaker et al. (2010a) found a significant association of sub-dimensions of the Social Cognition and Object Relations Scale (SCORS; Westen et al., 1991) that can be considered as a measure of psychic structure, to MAS-A ‘mastery.’ Due to a missing link of MAS-A to established measures of metacognition, Bröcker et al. (2017) further proposed a possible proximity of these concepts.

Theoretically, the acquisition of “full” synthetic metacognitive abilities might correspond to “reflexive competence” as a decisive step in the development of mentalizing capacities (Fonagy et al., 2002). From this stance, abilities of psychic structure that evolve during childhood are supposed to be interwoven with the emergence of mental state reflection and might shape actual metacognitive performance. Based on empirical findings and theoretical considerations, it was firstly hypothesized that metacognition and psychic structure would explain significant shares of variance in psychosocial functioning, beyond basic neurocognition and psychopathology. Considering that sub-dimensions of psychic structure reflect internalized representations of the self, others and unconscious attachment patterns that might set the frame for the development and actual performance of metacognition, it was further assumed that psychic structure underlies or mediates the impact of metacognition on functioning.

## Materials and Methods

The study was approved by the ethics committee of the Charité Universitätsmedizin Berlin. All subjects gave fully informed consent and were included in the baseline sample of the ongoing study “Modified Psychodynamic Psychotherapy for Patients with Schizophrenia – a Randomized-Controlled Efficacy Study” (MPP-S; ClinicalTrials.gov-ID: NCT02576613).

### Participants

Participants were 52 male and 48 female outpatients, aged between 19 and 63 years, fulfilling diagnostic criteria for schizophrenic or schizoaffective disorders according to the Diagnostic and Statistical Manual of Mental Disorders (DSM-IV-TR; American Psychiatric Association, 2000). Diagnoses were confirmed by a board certified psychiatrist with help of axis I of the German structured clinical interview for DSM-IV (SCID-I; Wittchen et al., 1997). Mean education levels were 15.12 (±3.13) years. Characteristics of illness and neuropsychological data are presented in Table 1. Medication protocols were as follows: Unmedicated: *n* = 9; combination of atypical and conventional neuroleptica: *n* = 7; exclusively atypical neuroleptica: *n* = 78; exclusively conventional neuroleptica: *n* = 4; exclusively mood stabilizer/antidepressants: *n* = 2; neuroleptica and additional mood stabilizer/antidepressants: *n* = 25. WHO Defined Daily Doses (World Health Organization Collaborating Centre for Drug Statistics Methodology, 2018) were calculated and provided in Table 1.

**TABLE 1:** Characteristics of illness and neuropsychological data (*N* = 100^a^).

	*M (SD)*
Illness duration (y)	13.10 (±9.31)
Number of hospital stays	4.88 (±5.14)
Age of onset (y)	25.02 (±7.85)
Current medication:	
WHO-DDD	1.20 (±1.40)
SGA	1.06(±0.98)
FGA	0.14(±0.67)
AVLT^(1–5)^	9.11 (±2.56)
WST-IQ	105.48 (±13.30)

**M* = mean; *SD* = standard deviation; y = years; WHO-DDD = defined daily doses of the World Health Organization; SGA = second generation antipsychotics; FGA = first generation antipsychotics; AVLT^(1–5)^ = Auditory Verbal Learning Test; mean score of the five initial presentations; WST-IQ = verbal IQ. ^*a*^AVLT^(1–5)^ with *N* = 97.*

### Instruments

#### Clinical Interview

A *semi-structured clinical interview* was conducted and evaluated, each by two investigators from a trained pool of clinical psychologists (Master’s degree) and psychiatrists. It lasted between 45 and 60 min and focused on various aspects: (1) psychopathology, (2) psychosocial functioning (everyday life, vocational capacities, interpersonal competencies) as well as (3) metacognition and psychic structure. The third and largest part of the interview aimed at eliciting emotionally relevant episodes in patient’s life and disease biography, in order to encourage reflectivity processes and assess metacognitive capacities as they arise naturally. Following rating principles of Lysaker et al. (2005), this passage was marked by non-directive conversation and an open attitude toward the subjective view of the interviewed persons. Specific questions aimed at evaluating higher reflecting processes if basic capacities had surfaced before (e.g., “Do you have any idea why being close to XY made you anxious?”) as well as to elicit more information about someone’s ability to decode other mental states (“How would your friend describe you?”, “What was her intention behind it?”). These more complex questions were only asked after a concrete scenario had been explored and core affects and possible cognitions had been successfully named (Dimaggio et al., 2015). Relevant episodes could either date back to early biography or belong to the present, depending on the actual capacities of the patients. It was particularly important to focus on relationships and interpersonal behavior in order to rate sub-dimensions of psychic structure, complying with OPD rating principles (OPD-2 Task Force, 2014). For this reason, countertransference was also taken into account and discussed afterward. Raters gained interview experiences during a pilot-study (Bröcker et al., 2017) and furthermore attended in-training sessions for the applied instruments (i.e., MINI-ICF and OPD workshops).

#### Clinical Ratings (Based on the Interview)

The *Metacognition Assessment Scale-Abbreviated* (MAS-A; Lysaker et al., 2005, 2010a) is an expert-rating of synthetic forms of metacognition as they arise in open narratives about life and illness that was recently translated into German (MAS-A-G; Bröcker et al., 2017).

It is based on the Metacognition Assessment Scale (MAS; Semerari et al., 2003, 2007), a tool to assess frequency of metacognitive acts within psychotherapy transcripts. MAS-A follows its main construction of sub-dimensions, but was adapted to interview situations and particularly emphasizes increasing complexity of single metacognitive acts (Lysaker et al., 2020). Four implied sub-dimensions represent semi-independent metacognitive capacities, namely self- and other reflection (S-Scale: “Understanding of One’s Mind,” O-Scale: “Understanding of Other’s Mind), perspective-taking (D-Scale: “Decentration”) and the overall capacity to integrate and use metacognitive knowledge to master intrapsychic and interpersonal challenges adopting a psychologically minded stance (M-Scale: “Mastery”). Each subscale consists of an array of items representing increasing complex metacognitive capacities; every function (e.g., S4: “The subject is able to define and distinguish his own emotional states”) is rated as “1” (fully present), “0.5” (partly present) or “0” (not present).

An overview of our sample’s distribution according to each subscale as well as the total scale is given in Table 2. MAS-A was widely used in various studies with samples of individuals with schizophrenia spectrum disorders in the last decade, replicating good inter-rater reliabilities (Lysaker et al., 2005; Lysaker and Dimaggio, 2014), equally with a German translation that was applied here (MAS-A-G; Bröcker et al., 2017).

**TABLE 2:** Characteristics of metacognition (MAS-A) with *N* = 100.

	Min	Max	*M* (SD)
S-scale	1.50	9.00	6.46 (±1.72)
O-scale	1.00	7.00	4.30 (±1.24)
D-scale	0.00	3.00	1.83 (±0.83)
M-scale	0.50	9.00	5.64 (±1.78)
Total scale	4.50	27.00	18.22 (±4.88)

*MAS-A = Metacognition Assessment Scale-Abbreviated; Min = minimum; the lowest scoring; Max = maximum; the highest scoring; *M* = mean; *SD* = standard deviation; S-Scale = understanding of one’s mind; O-Scale = understanding of other’s mind; D-Scale = decentration; M-Scale = mastery.*

The *Level of Structural Integration Axis* as one out of five axes *of Operationalized Psychodynamic Diagnosis* (OPD-LSIA; OPD-2 Task Force, 2014) in its original German form was considered as a measure of psychic structure. OPD-LSIA has a psychodynamic background and comprises an individual’s emotional and cognitive core competencies that were shaped in early interactions and are considered to be elementary to maintain intrapsychic as well as interpersonal homeostasis. It differentiates four basic dimensions that are further defined with regard to the self as well as with regard to interaction with external objects, resulting in overall eight sub-dimensions: (Reflexive) perception (self-perception vs. object perception), regulation (self-regulation vs. regulation of object relationship), communication (internal communication vs. communication with the external world) and attachment (attachment to internal vs. attachment to external objects). Each of these sub-dimensions is described with help of three characteristic items (e.g., for self-regulation: affect tolerance, impulse control, regulation of self-esteem), each of which is then rated as either 1 (“high level of structural integration”), 2 (“moderate level of structural integration”), 3 (“low level of structural integration”) or 4 (“disintegration”), before a mean value per sub-dimension is calculated, with higher values indicating greater difficulties. For this study, an overall sum score was calculated and included in statistical analyses. An overview of OPD-LSIA sub-dimensions and our sample’s distribution is given in Table 3. The instrument proved good psychometric properties (Cierpka et al., 2007; Benecke et al., 2009; Zimmermann et al., 2012; OPD-2 Task Force, 2014).

**TABLE 3:** Characteristics of psychic structure (OPD-LSIA) with *N* = 100.

	Min	Max	*M* (*SD*)
Self perception	1.17	4.00	2.50 (±0.63)
Object perception	1.33	4.00	2.96 (±0.61)
Self-regulation	1.67	4.00	2.72 (±0.47)
Regulation of relationship	1.17	4.00	2.71 (±0.57)
Internal communication	1.00	4.00	2.65 (±0.64)
External communication	1.33	3.67	2.56 (±0.60)
Attachment to internal objects	1.33	4.00	2.92 (±0.56)
Attachment to external objects	1.67	4.00	3.00 (±0.59)
OPD-LSIA Mean value	1.75	3.90	2.75 (±0.48)
OPD-LSIA Total Scale	14.00	31.17	22.01 (±3.81)

*OPD-LSIA = Level of Structural Integration Axis of Operationalized Psychodynamic Diagnosis; Min = minimum. the lowest scoring; Max = maximum. the highest scoring; *M* = mean; *SD* = standard deviation; Mean Value = sum value/number of subscales; Total scale = sum score.*

A five-factor model of the *Positive and Negative Syndrome Scale* (PANSS; Kay et al., 1987) including positive and negative symptoms, cognition, depression/anxiety and excitement/hostility (Citrome et al., 2011) was applied next to the *Scale for the Assessment of Negative Symptoms* (SANS), the *Scale for the Assessment of Positive Symptoms* (SAPS) (Andreasen, 1982; Andreasen and Olsen, 1982; Andreasen et al., 1995) and the German version of the *Calgary Depression Scale for Schizophrenia* (CDSS-G; Müller et al., 1999), in order to assess psychopathology. Characteristics of all dimensions of psychopathology are provided in the Supplementary Table S1.

A short version of *the International Classification of Functioning, Disability and Health* (MINI-ICF-APP; Linden et al., 2009), an expert-rating considering capacities *to participate* in society, provided by the World Health Organization, was applied in order to measure psychosocial functioning. It consists of 13 sub-categories, namely adjustment to rules and routines, planning and structuring of tasks, flexibility and adaptability, application of professional competencies, decision-making and judgment abilities, sustainability, power of self-assertion, small talk skills, behavior in groups, familiar and intimate relationships, spontaneity, self-care/hygiene and ability to use public transports, each of which was rated on a five-point Likert-scale (0 = no impairment, 1 = mild impairment, 2 = moderate disability, 3 = severe disability, 4 = total disability). Following rating principles, interview information and behavior during the interview session were considered and the assessment was based on a comparison between the actual vs. the premorbid state, thus indicating *disease-related changes.* A sum score was built for further calculations.

#### General Cognitive Functioning

A vocabulary test (WST; Schmidt and Metzler, 1992) was applied to estimate premorbid verbal intelligence. A mean score of the five initial presentations [AVLT^(1–5)^] of the *Auditory Verbal Learning Test* (AVLT; Heubrock, 1992) was used in order to assess verbal memory and learning.

### Procedure

Participants were recruited with support of psychiatric hospitals, ambulant psychiatrists, psychotherapists as well as members of the psychosocial care system (e.g., social workers) in Berlin and Brandenburg, Germany. Inclusion criteria were confirmed during a screening interview that could either be performed face to face or on the phone, before patients gave fully informed consent. MAS-A, OPD-LSIA, psychopathology (SAPS, SANS, PANSS, CDSS) and MINI-ICF scores were rated consensually based on the above described clinical interview. After a break, AVLT and WST were conducted. Average duration of one appointment was two hours.

### Statistical Analysis

#### Principal Component Analysis

A principal component analysis (PCA) was performed to reduce data of included psychopathology measures, namely PANSS, SANS, SAPS and CDSS. It also aimed at integrating detailed information considering specific symptom dimensions from different instruments (e.g., various subordinate aspects of negative or depressive symptoms) into one model for further calculations. PCA was conducted on the level of validated factor solutions of the respective measures and included altogether 16 subscales. Regarding PANSS, the five-factor model of Citrome et al. (2011) was chosen, as (a) a five-factor solution is broad consensus (Wallwork et al., 2012), (b) it does not allow for double loadings, (c) it is an inclusive model (does not exclude any of the 30 items) and (d) it is comparable with other prominent factor models (van der Gaag et al., 2006). The initial five subscales of SANS and SAPS were included together with one sum score of CDSS. PCA was conducted with orthogonal rotation (varimax), break-off criterion was eigenvalue > 1 (Kaiser’s criterion). Preconditions for PCA were checked by means of Kaiser-Meyer-Olkin measure of sampling adequacy, which was ‘good’ (KMO = 0.78), supported by acceptable individual KMO values starting from 0.56 (Field, 2018) and Bartlett-test of sphericity [χ^2^(120) = 889.63, *p* < 0.001]. For further calculations, (standardized) Anderson-Rubin scores were computed (Distefano et al., 2009).

PCA identified four factors accounting for 67.96% of the total variance. An overview of the extracted factors and factor loadings after varimax rotation, is given in Table 4. Factor 1 included dimensions representing *negative or cognitive symptoms* (36.63%), Factor 2 described *positive symptoms* (+14.52%), Factor 3 *depressive symptoms* (+9.47%) and Factor 4 subsumed *disorganization* or *excitement* (+7.35%).

**TABLE 4:** Rotated component matrix^a^.

	Factor 1: Negative symptoms (5.86)^b^	Factor 2: Positive symptoms (2.32)	Factor 3: Depressive symptoms (1.52)	Factor 4: Disorganization or excitement (1.18)
SANS_alogia	**0.836**	0.077	0.124	0.211
PANSS_negative	**0.761**	0.223	0.429	−0.093
SANS_affective flattening	**0.752**	0.088	0.226	−0.147
SANS_attention	**0.590**	0.244	0.147	0.333
PANSS_cognition	**0.580**	0.432	0.368	0.242
PANSS_positive	0.201	**0.882**	0.077	0.129
SAPS_delusion	0.083	**0.848**	0.277	0.173
SAPS_hallucinations	0.166	**0.774**	0.136	−0.095
CDSS_sum	0.145	0.096	**0.895**	0.061
PANSS_depression	0.257	−0.026	**0.842**	−0.032
SANS_apathy	0.181	0.244	**0.732**	−0.028
SANS_anhedonia	0.311	0.331	**0.528**	0.027
PANSS_excitement	−0.184	0.133	−0.007	**0.758**
SAPS_inappropriate affect	0.103	−0.081	−0.070	**0.678**
SAPS_positive formal thought disorder	0.119	0.566	−0.079	**0.615**
SAPS_bizarre behavior	0.334	0.094	0.181	**0.594**

*SANS = Scale for the Assessment of Negative Symptoms; SAPS = Scale for the Assessment of Positive Symptoms; PANSS = Positive and Negative Syndrome Scale: five-factor solution of Citrome et al. (2011); CDSS = Calgary Depression Scale for Schizophrenia. ^*a*^Varimax rotated: 5 iterations. ^*b*^Eigenvalues. Factor loadings > 0.50 are indicated in bold typing.*

#### Regression Analyses

**FIGURE 1:**
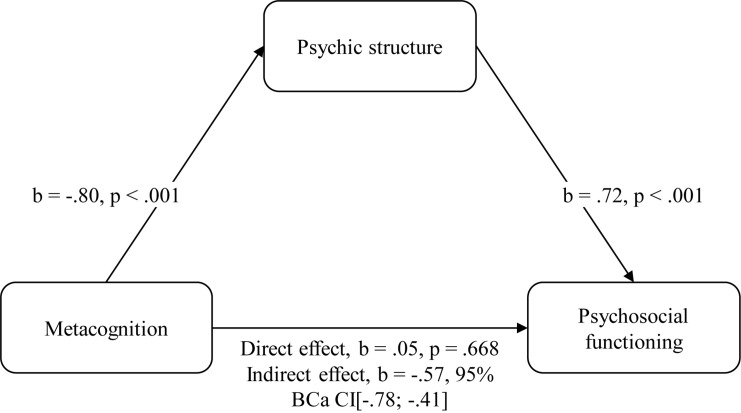
Mediation analysis. Metacognition has an indirect effect via psychic structure on functioning. The confidence interval for the indirect effect is a BCa bootstrapped CI based on 5000 samples.

Two hierarchical regression analyses were performed with psychosocial functioning (MINI-ICF SUM) as the outcome variable. Cognitive variables entered first (AVLT, WST; model 1), followed by adding symptom factors 1–4 (model 2), metacognition measured with the MAS-A (model 3a) and psychic structure measured with the OPD-LSIA (model 3b). Furthermore, a mediation analysis was performed to explore whether psychic structure mediated the potential impact of metacognition on functioning (see Figure 1).

Metacognition and psychic structure were included in separate regressions to deal with multicollinearity effects (*r* = −0.80, *p* < 0.001). A correlational table considering all included predictors as well as the outcome variable is provided in the Supplementary Table S2 with Bonferroni corrected significance levels to account for an accumulation of type I error due to multiple testing (Shaffer, 1995). As Shapiro–Wilk test indicated non-normally distributed data for psychopathology (F1-4), with substantial skewness (>1) for F1 and F4, bias-corrected and accelerated (BCa) bootstrap confidence intervals (with 5000 samples) were computed to corroborate Pearson correlation coefficients (Banjanovic and Osborne, 2016). BCa confidence intervals were also reported for regression weights in order to provide a consistent presentation of results and to deal with single violations of homoscedasticity. The Akaike information criterion (AIC; Akaike, 1973) as a measure of goodness-of-fit was further calculated to correct for increasing model complexity. All variables were z-standardized before statistical analyses, which were conducted using SPSS Statistics for MAC version 25.0. For the mediation analysis, the PROCESS macro was installed (Hayes, 2019).

## Results

### Impact of Psychic Structure and Metacognition on Functioning

Negative, positive as well as depressive symptoms explained a significant amount of variance in functioning overall models, whereas disorganization as well as cognitive variables were no significant predictors. Thus, only model 2-4 displayed significance, with model 3b (including psychic structure) explaining a maximum of 77% of variance in the outcome [adjusted *R*^2^ = 0.75, *F*(1,89) = 16.62, *p* < 0.001]. Metacognition (*b* = −0.20, 95% BCa CI [−0.36; −0.05], *p* = 0.029) as well as psychic structure (*b* = 0.33, 95%BCa CI [0.18;0.50], *p* < 0.001) significantly contributed beyond cognition and psychopathology, with psychic structure explaining more variance (Model 3b with psychic structure: Δ *R*^2^ = 0.04, *p* < 0.001 vs. Model 3a with metacognition: Δ *R*^2^ = 0.01, *p* = 0.029, each compared to Model 2). Changes in *R*^2^ could be supported by decreasing AIC values. An overview is presented in Table 5.

**TABLE 5:** Multiple linear regressions predicting MINI-ICF psychosocial functioning (*N* = 100^a^).

	*b*	95% BCa CI^b^	*SE* b^b^	*ß*	*t*	*p*	AIC
**Model 1**							−3.74
Constant	−0.03	[−0.22;0.16]	0.10		−0.30	0.768	
AVLT^(1–5)^	−0.21	[−0.42;0.03]	0.10	−0.21	−1.99	0.049	
WST-IQ	0.01	[−0.23;0.24]	0.12	0.01	0.08	0.936	
**Model 2**							−116.08
Constant	0.01	[−0.10;0.13]	0.06		0.19	0.853	
AVLT^(1–5)^	0.02	[−0.09;0.15]	0.06	0.02	0.34	0.737	
WST-IQ	0.02	[−0.12;0.15]	0.06	0.02	0.27	0.788	
Factor 1	0.41	[0.28;0.57]	0.07	0.39**	6.54	0.000	
Factor 2	0.46	[0.34;0.58]	0.06	0.46**	8.26	0.000	
Factor 3	0.64	[0.51;0.77]	0.06	0.65**	11.63	0.000	
Factor 4	0.11	[−0.02;0.23]	0.06	0.11	1.89	0.062	
**Model 3a**							−119.28
Constant	0.00	[−0.11;0.11]	0.06		0.02	0.986	
AVLT^(1–5)^	0.02	[−0.09;0.16]	0.06	0.02	0.33	0.745	
WST-IQ	0.06	[−0.08;0.19]	0.06	0.06	0.95	0.346	
Factor 1	0.31	[0.16;0.47]	0.08	0.30**	4.13	0.000	
Factor 2	0.35	[0.21;0.50]	0.08	0.35**	4.67	0.000	
Factor 3	0.64	[0.51;0.76]	0.06	0.65**	11.79	0.000	
Factor 4	0.04	[−0.13;0.16]	0.07	0.04	0.58	0.565	
MAS-A SUM	−0.20	[−0.37;−0.04]	0.09	−0.20^∗^	−2.21	0.029	
**Model 3b**							−130.69
Constant	−0.01	[−0.11;0.10]	0.05		−0.10	0.919	
AVLT^(1–5)^	0.00	[−0.11;0.12]	0.06	0.00	0.00	0.999	
WST-IQ	0.02	[−0.10;0.14]	0.06	0.02	0.31	0.758	
Factor 1	0.25	[0.12;0.39]	0.07	0.24**	3.66	0.000	
Factor 2	0.26	[0.11;0.41]	0.08	0.26**	3.72	0.000	
Factor 3	0.57	[0.45;0.70]	0.06	0.58**	10.59	0.000	
Factor 4	−0.01	[−0.14;0.11]	0.06	−0.01	−0.10	0.922	
OPD-LSIA SUM	0.33	[0.18;0.50]	0.08	0.33**	4.08	0.000	

*R*^2^ = 0.04 for step 1 (*p* = 0.118); Δ *R*^2^ = 0.68 for step 2 (*p* < 0.001); Δ *R*^2^ = 0.01 for step 3 from Model 2 to Model 3a (*p* = 0.029); Δ *R*^2^ = 0.04 for step 4 from Model 2 to Model 3b (*p* < 0.001). *p < 0.05, **p < 0.01. MINI-ICF = International Classification of Functioning, Disability and Health (short version); b = regression coefficient; BCa CI = confidence interval; SE = standard error; ß = standardized regression weight; df = degrees of freedom; AIC = Akaike’s information criterion (goodness-of-fit); AVLT^(1–5)^ = Auditory Verbal Learning Test: mean score of the five initial presentations; WST-IQ = verbal IQ; Factor 1–4 = symptom factors: “negative or cognitive symptoms,” “positive symptoms,” “depressive symptoms,” “disorganization/excitement”; MAS-A sum = sum score of the Metacognition Assessment Scale – Abbreviated; OPD-LSIA SUM = sum score of the Level of Structural Integration Axis of Operationalized Psychodynamic Diagnosis. ^*a*^AVLT^(1–5)^ with *N* = 97. ^*b*^Confidence intervals and standard errors per BCa-Bootstrapping with 5000 BCa samples.

### Psychic Structure Mediates the Impact of Metacognition on Functioning

Mediation analysis showed that a direct effect of metacognition on functioning disappeared when including psychic structure as a mediator variable (*b* = 0.05, 95% BCa CI [−0.19; 0.30], *p* = 0.668). A significant indirect effect was found (*b* = −0.57, 95% BCa CI [−0.78; −0.41]) which means that alterations in psychic structure mediated the impact of alterations in metacognition on alterations in functioning. The full mediation model is shown in Figure 1.

## Discussion

As a continuation of our previous work, a German translation of the Metacognition Assessment Scale-Abbreviated (MAS-A-G; Bröcker et al., 2017), a tool to assess synthetic aspects of metacognition (MAS-A; Lysaker et al., 2005, 2010a), was concurrently applied with the operationalized psychoanalytic construct of psychic structure (OPD-LSIA; OPD-2 Task Force, 2014). It was hypothesized that both constructs would contribute incremental value to explained variance in functioning beyond psychopathology and cognition. It was further explored whether psychic structure would underlie and therefore mediate the impact of metacognition on functioning. Psychopathology explained a high amount of variance in function, while cognition did not. When testing two concurrent models which respectively included (a) metacognition and (b) psychic structure beyond cognition and psychopathology, the latter explained slightly more variance in the outcome. Furthermore, the expected mediation effect was found.

Metacognition contributed significantly beyond cognition and symptoms. This finding is in line with a robust body of evidence associating metacognitive abilities with psychosocial functioning in patients with psychoses (meta-analysis by Arnon-Ribenfeld et al., 2017; Lysaker et al., 2018b; Gagen et al., 2019) and proposed mediating models prompting a decisive role of metacognitive “mastery” in explaining predictive effects based on neurocognition (Lysaker et al., 2010b). Results are also in accord with research confirming a superior impact of higher-order cognition on functioning (Couture et al., 2006; Green et al., 2019; Halverson et al., 2019), as metacognition and social cognition might share substantial overlap. In contrast to social cognition, metacognition has not been explicitly included in the provided definitions of the “Measurement and Treatment Research [group] to Improve Cognition in Schizophrenia” of the National Institute of Mental Health (MATRICS; Green et al., 2005) and a clear distinction was suggested elsewhere, as dimensions of both concepts showed varying association patterns with different outcome measures and loaded empirically on different factors (Lysaker et al., 2013a; Hasson-Ohayon et al., 2015). Results of a recent, extensive meta-analysis (Halverson et al., 2019), examining predictions based on neurocognitive and social cognitive domains, pointed out that a high amount of variance in functioning might still remain unexplained. Synthetic metacognition might provide additional value at this point (Lysaker et al., 2013a). It was argued that social cognitive domains rather capture basic or discrete reflexive moments of self and other reflection within circumscribed laboratory settings, whereas synthetic metacognition is defined *per se* as a continuous integrating and contextualizing process (Hasson-Ohayon et al., 2018; James et al., 2018).

There is no doubt that the development of the Metacognition Assessment Scale-Abbreviated can be considered a milestone in metacognition research; however, it primarily concentrates on descriptive and evaluating aspects of abilities, also in the course of psychotherapy (Lysaker et al., 2018b). Even though it acknowledges theories of early development, its focus is on reflecting actual and future interpersonal challenges. It thus abstains from looking more deeply into the inner dispositions that might modulate an individual’s capacity to integrate and synthesize discrete reflexive sequences. From a psychoanalytic stance, one could argue that internalized object relations are inseparably interwoven with the continuous formation of representations of actual social relationships and contexts (OPD-2 Task Force, 2014). These might serve as working schemas and implicitly shape actual metacognitive performance as measured with the MAS-A via (co)transference. Although early object relations were considered in metacognition research, they were not associated with functional outcome and were therefore interpreted as “reflect[ing] the participant’s own internal world instead of the nuances of the actual people with which they confront during real-life interactions” (Lysaker et al., 2010a), illustrating that the theoretical framework of the psychoanalytic approach is distinct from the metacognitive approach.

In the present work, the psychodynamic key concept of psychic structure – explicitly considering internalized object relations based on early child-caregiver interactions – was included in a concurrent model, and results showed a slight advantage in explaining incremental variance in psychosocial functioning compared to cognition, psychopathology and metacognitive abilities. These findings might indicate an additional value of the concept of psychic structure in both the prediction and treatment of deficits in psychosocial function in psychotic disorders. For the above-mentioned reasons, psychic structure was further explored as underlying or mediating variable of the impact of metacognition on function. Even if results can only be considered preliminary at this point, they might be a relevant starting point to further investigate metacognition together with internalized object relations that are anchored in psychoanalytic theory. Our finding is in line with research indicating a role of inner working models of attachment for recovery from psychosis as well as a prerequisite to profit from therapeutic interventions (Gumley et al., 2014; Debbané et al., 2016). It is also worthwhile to mention that psychic structure as measured with the OPD-LSIA refers to level of personality functioning (Zimmermann et al., 2012), that has been additionally considered in recent development of diagnostic systems (DSM-5; American Psychiatric Association, 2013). However, research on how this might relate to other established measures of more general psychosocial functioning (Buer et al., 2019) or the concept of mentalizing (Zettl et al., 2019) emerges only now.

Interventions that tackle mental state reflection entered into various therapeutic approaches, with some of them considering underlying interpersonal schemas. While the Metacognitive Interpersonal Therapy (MIT; Dimaggio et al., 2017; Popolo et al., 2019) focuses on metacognitive acts belonging to maladaptive schemas that interfere with the accomplishment of actual life motives, the Metacognition-Oriented Social Skills Training (MOSST; Ottavi et al., 2014; Inchausti et al., 2018) included an interpersonal level to classic SST group interventions, continuously exploring underlying processes between group members in terms of feelings and cognitions. While both approaches consider intersubjectivity and biographically shaped schemas, they work with conscious and explicit interpersonal knowledge from the beginning of the therapy. In contrast to that, work on psychic structure implies that maladaptive interpersonal behavior is partly unconscious and manifests itself in interpersonal interactions, as presumed in psychoanalytically rooted approaches like Mentalization-based therapy (MBT; Fonagy and Luyten, 2009) that has now been adjusted to work with psychosis (Brent, 2015; Weijers et al., 2016). One main characteristic of the Metacognitive Reflection and Insight Therapy (MERIT; Lysaker and Klion, 2017), a modern metacognitive approach to psychosis, is that interventions can be integrated within different therapeutic orientations (Lysaker et al., 2020). With regard to this attempt, it might be of particular interest to more specifically investigate a possible interplay of synthetic metacognition with underlying unconscious mechanisms based on internalized object representations. It might also be valuable to explore to what extent improving metacognition might be used to compensate structural deficits or – in the course of therapy – enable an individual to tackle them more deeply.

MERIT was developed along with the instrument MAS-A and its established link to functioning. Even though this scientifically relevant starting point, multidisciplinary exchange during its origin, and case studies are encouraging (Lysaker et al., 2018b, 2019b, 2020), randomized-controlled pilot-studies could, until now, not verify the assumed predictive effects on functioning and work readiness as secondary outcomes (de Jong et al., 2019). More discrete metacognitive interventions (Moritz et al., 2014, 2016) as well as cognitive behavioral approaches to non-affective psychosis (Jauhar et al., 2014) primarily define their strength in modulating cognitive biases, thus focus on an improvement of metacognitive *monitoring* and a decrease of positive symptoms, whereas beneficial effects on social functioning are still debated (Laws et al., 2018). Unfortunately, there is a blatant lack of empirical evidence according to current scientific standards regarding the treatment effects of modern psychodynamic approaches to psychosis. This is also mirrored by missing recommendations of such interventions in relevant treatment guidelines. Nevertheless, results of a naturalistic, quasi-randomized study of a manualized, psychosis-specific, psychodynamic psychotherapy showed significant positive effects on psychosocial functioning after 2 years of treatment compared to treatment as usual (Rosenbaum et al., 2012), whereas this effect was not sustained at 5-year follow up (Harder et al., 2014). Moreover, a growing body of literature integrating relevant psychodynamic concepts emerged (Harder and Folke, 2012; Lempa et al., 2016) and RCTs investigating psychodynamic approaches for patients with psychoses are currently conducted (Weijers et al., 2016; MPP-S; ClinicalTrials.gov-ID: NCT02576613). However, observing structural levels of integration of patients diagnosed with non-affective psychosis, psychodynamic techniques should be modified for an application to this patient group.

In the following, an insight into theories that underlie modern approaches will be given. As the given results indicate importance of interventions that target psychic structure, theories might be of interest and should be further investigated in upcoming studies. From a theoretical stance, psychodynamic approaches to psychosis could be considered as a process of different stages or therapeutic stances and integrate a specific, *implicit* psychotherapeutic work that must precede and accompany any “classical,” *explicit* interventions like biographical reconstruction or interpretation (Lempa et al., 2016). *Implicit* procedures mainly take place within the psychotherapeutic relationship with the aim to attenuate interpersonal fears and to configure an interpersonal field, laying the groundwork for further, reflection-based interventions. This primary therapeutic focus can only be understood within a theoretical framework of psychotic disintegration as the result of an existential tensional state between self-related vs. object-related tendencies – referred to as “dilemmatic” (Benedetti, 1998; Mentzos, 2015). Presuming an incomplete separation of inner representations of self and important others, patients cannot be close to others without being threatened by loss of self-coherence or fragmentation or, conversely, have to withdraw completely from the social world in order to protect their own identity. In contrast to neurotic conflicts, *dilemmatic states* are characterized by a lack of potential to mentally *represent* this inner field of tension. As a result, the affected person can get *existentially threatened* in (emotionally relevant) interpersonal encounters, and without other means of regulation, psychosis might become a last resort to maintain a – although distorted – contact with the social world. An array of implicit therapeutic interventions attempt to tackle this fundamental interpersonal disturbance and to foster an exemplary, modifying interpersonal experiences in “real-time” within the therapeutic relationship – corresponding to Stern’s concept of “moving along” or “present moments” (Stern, 1985, 2004). This decisive, implicit regulation of interpersonal closeness versus distance is the first precondition for recovering the capacity to represent and to mentalize inner and outer experiences, and to reflect on them from a metacognitive position – thus, for the post-maturing of structural capacities (Mentzos and Münch, 2010; Lempa et al., 2016).

The patient’s abilities to sustain a sense of identity during a therapeutic encounter and to take a reflexive stance within a working relationship might wax and wane, depending on dialogue topic or situation, but may be regained in the course of treatment. This presupposes a permanent consideration of the implicit interpersonal scene that can often be deduced only from the therapist’s own inner reactions. Current metacognitive approaches, like MERIT (Lysaker and Klion, 2017), already include facets of implicit work without fully conceptualizing it. A recent publication is compatible with psychodynamic practice as it pointed out the need to supervise (co)transference phenomena in response to fragmentation (Lysaker et al., 2019a). Working with co- and counter-transference has a long psychoanalytic tradition and particularities in response to psychotic states were elaborated and emphasized in modern approaches (Mentzos, 2015; Lempa et al., 2016). Conceptualizations of implicit work with patients with psychoses should therefore focus on patients’ competence to maintain a sense of identity on the one hand and on therapists’ personal experiences and regulative skills in therapeutic relationships on the other. We would propose that a preferential focus on implicit or explicit interventions should sensitively depend on the current capacities of the patient.

As expected, psychopathology – apart from disorganization – explained a high amount of variance in functioning (Ahmed et al., 2015), whereas variables of general cognition, contrary to our expectations, did not contribute significantly. These results are opposing to recent findings (Green et al., 2000, 2004; Ventura et al., 2013) and might be explained by a different operationalization of psychosocial functioning in the present study. Application rules of MINI-ICF-APP – a rating of disturbances of activity and participation devised by the World Health Organization - used here, precisely require *to compare* the actual to the premorbid level of functioning and only to weight noticeable changes. In contrast to that, common functional outcome measures tend to focus on the current state with only limited regard to former intellectual, vocational or interpersonal attainments. Cognitive dimensions like crystalline intelligence (measured with the WST) as rather stable capacities, partly independent of the course of disease, could thus be associated with general or absolute limitations in functioning, but do not necessarily account for disease-related *changes*. Disorganization, on the other hand, might be considered as a state-related marker of acuity that might thus equally not be a stable predictor for sustained outcome changes.

There are several limitations to this study: The investigated sample had on average moderate to low integration levels of psychic structure, which were relatively high compared to the only other study of individuals with non-affective psychoses using the OPD (Uzdawinis et al., 2010), and were closer to ratings of personality disorders (Doering et al., 2014). This finding might partly be explained by the observation that patients with psychosis show particular characteristics of psychic structure and that sometimes “disintegrated” as well as better integrated aspects of the same dimension can exist simultaneously at the same time. Psychotic experiences can be limited to a few crucial aspects of life, while in other areas non-psychotic ways of functioning prevail. Considering that structure according to OPD-2 should be rated regarding the previous two years, during which healthy as well as illness periods might occur, specific structural capacities can be obfuscated during acute exacerbations but deficits do not necessarily persist during periods of remission. A revised version of the OPD might therefore allow for ratings of structural flexibility over time and the pervasiveness of psychotic reactions across all important areas of life.

Even if MAS-A and OPD-LSIA rating principles were trained and strictly respected, all expert-ratings were applied to the same interview, and consensus was obtained by the same two investigators. Variables on the rater level, like experience and extent of psychoanalytic educational background, could thus have influenced the results and a potential rater bias could not be excluded. The correlation coefficient between MAS-A and OPD-LSIA was unexpectedly rather high and prevented inclusion into a common regression model. Therefore, findings of the present study must be considered as preliminary and replicated in independent samples, including healthy controls as well as other clinical populations with different levels of structural integration. Future clinical studies might include comparisons between psychodynamic and synthetic metacognitive approaches in terms of specific interventions, as well as multi-method designs, relative vs. absolute measures of psychosocial functioning and further relevant outcome measures like suicidality (Hutton et al., 2019).

Finally, considering metacognition and psychic structure as potential indicators of change during psychotherapy that can only be observed after an adequate amount of time (Leuzinger-Bohleber et al., 2019), longitudinal study designs are needed in addition to the cross-sectional analyses conducted here.

In summary, structural integration explained more variance than synthetic metacognition in psychosocial functioning of individuals diagnosed with non-affective psychosis, and mediated its linkage. Results of the present work thus indicate some additional value of the psychodynamic construct of psychic structure in terms of the prediction of psychosocial capacities that might also guide psychotherapeutic strategy. Even if particularities of the psychoanalytic approach were highlighted, we equally value the metacognitive viewpoint and hope that this work will contribute to interdisciplinary understanding and communication and to ultimately determine “what works for whom and when” in a patient group woefully neglected regarding their psychotherapeutic needs (Rosenbaum and Harder, 2007; Hamm and Lysaker, 2016; Lysaker et al., 2018a). From the authors’ perspective this might include a theoretically informed and subtle consideration of complementary implicit therapeutic interventions.

## Data Availability Statement

The datasets generated for this study are available on request to the corresponding author.

## Ethics Statement

The studies involving human participants were reviewed and approved by ethics committee of the Charité Universitätsmedizin Berlin. The patients/participants provided their written informed consent to participate in this study.

## Author Contributions

GL, DH, and CM are responsible for the original study design from which data was taken. A-LB, SB, FS, SJ, GB, JF, IG, and CM collected the data. A-LB and CM performed the statistical analyses and wrote the draft with theoretical input of GL and DH and reviewing by SB, FS, and SJ.

## Conflict of Interest

The authors declare that the research was conducted in the absence of any commercial or financial relationships that could be construed as a potential conflict of interest.
