# Supplementary material for: Levels of Structural Integration Mediate the Impact of Metacognition on Functioning in Non-affective Psychosis: Adding a Psychodynamic Perspective to the Metacognitive Approach
Source: Front Psychol. 2020 Feb 21;11:269. doi: 10.3389/fpsyg.2020.00269 (PMC7047329; doi:10.3389/fpsyg.2020.00269)
Supplement: Supplementary file 1 [file Data_Sheet_1.PDF]

## Supplementary Tables

### Table S1: Psychopathology (*N*=100)

| | Min | Max | *M (SD)* |
|---|---|---|---|
| **PANSS** | | | |
| Total | 31 | 107 | 58.12 (±14.84) |
| Positive | 5 | 22 | 10.79 (±4.77) |
| Negative | 7 | 32 | 14.85 (±5.65) |
| Cognition | 9 | 31 | 15.93 (±4.79) |
| Excitement/hostility | 4 | 13 | 5.61 (±1.88) |
| Depression/anxiety | 5 | 24 | 10.93 (±4.01) |
| **SANS** | | | |
| Total | 0 | 72 | 22.73 (±14.95) |
| Affective flattening | 0 | 22 | 4.41 (±5.20) |
| Alogia | 0 | 16 | 2.04 (±3.69) |
| Avolition | 0 | 14 | 5.72 (±3.82) |
| Anhedonia | 0 | 22 | 9.34 (±6.11) |
| Attention | 0 | 9 | 1.22 (±2.29) |
| **SAPS** | | | |
| Total | 0 | 83 | 17.64 (±16.66) |
| Hallucinations | 0 | 25 | 4.31 (±6.22) |
| Delusions | 0 | 32 | 7.26 (±7.57) |
| Bizarre behavior | 0 | 10 | 0.84 (±1.82) |
| Thought disorder | 0 | 25 | 4.92 (±5.70) |
| Inappropriate affect | 0 | 3 | 0.31 (±0.75) |
| **CDSS** | | | |
| Total | 0 | 24 | 5.89 (±5.44) |

*Note.* Min = Minimum; Max = Maximum; *M* = Mean; SD = standard deviation; PANSS = Positive and Negative Syndrome Scale: five-factor solution of Citrome, Meng, & Hochfeld (2011); SANS = Scale for the Assessment of Negative Symptoms ; SAPS = Scale for the Assessment of Positive Symptoms; CDSS = Calgary Depression Scale for Schizophrenia.

**Table S2: Correlations[a] of MINI-ICF sum as the outcome variable with all considered predictors ($N^b$ = 100)**

| | MINI-ICF sum | AVLT $^{(1-5)}$ | WST-IQ | Factor 1 | Factor 2 | Factor 3 | Factor 4 | MAS-A sum | OPD-LSIA sum |
|---|---|---|---|---|---|---|---|---|---|
| MINI-ICF sum | 1 | -.21* [-.41;.01] | -.06 [-.29;.18] | .31** [.07;.50] | .43** [.23;.60] | .62** [.47;.75] | .05 [-.13;.23] | -.50** [-.64;-.32] | .66** [.55;.75] |
| AVLT $^{(1-5)}$ | | 1 | .34** [.14;.51] | -.32** [-.52;-.09] | -.10 [-.32;.13] | -.12 [-.31;.07] | .11 [-.15;.37] | .23* [.03,.41] | -.13 [-.35;.11] |
| WST-IQ | | | 1 | -.25* [-.46;-.02] | .02 [-.23;.26] | -.02 [-.22;.19] | .13 [-.04;.28] | .27** [.03;.50] | -.04 [-.27;.18] |
| Factor 1 | | | | 1 | -.06 [-.30;.18] | -.06 [-.27;.13] | -.06 [-.25;.13] | -.46** [-.64;-.24] | .36** [.14;.55] |
| Factor 2 | | | | | 1 | -.01 [-.24;.22] | -.06 [-.23;.13] | -.51** [-.65;-.32] | .54** [.40;.67] |
| Factor 3 | | | | | | 1 | -.02 [-.19;.18] | .01 [-.19;.24] | .17 [-.02;.35] |
| Factor 4 | | | | | | | 1 | -.26** [-.44;-.07] | .28** [.10;.45] |
| MAS-A sum | | | | | | | | 1 | -.79** [-.86;-.69] |
| OPD-LSIA sum | | | | | | | | | 1 |

*Note.* MINI-ICF = International Classification of Functioning, Disability and Health (short version); AVLT $^{(1-5)}$= Auditory Verba Learning Test: mean score of the five initial presentations; WST-IQ = verbal IQ; Factor 1-4 = symptom factors: „negative or cognitiv symptoms", "positive symptoms", "depressive symptoms", "disorganization/excitement"; MAS-A sum = sum score of the Metacognitio Assessment Scale – abbreviated; OPD-LSIA SUM = sum score of the Level of Structural Integration Axis of Operationalize Psychodynamic Diagnosis.

[a] Pearson correlation coefficients are supported by bootstrapped confidence intervals based on 5000 samples.

[b] AVLT $^{(1-5)}$ and WST-IQ with *N* = 97.

* $p<0.05$. ** $p<0.01$. Corrected p-value (Bonferroni) $p < 0.001$.

Values were z-standardized before analyses.
